# Childhood cancer and environmental integrity: a commentary and a proposal

**DOI:** 10.1590/S1518-8787.2017051006744

**Published:** 2017-03-29

**Authors:** Carlo Modonesi, Enrico Oddone, Celestino Panizza, Gemma Gatta

**Affiliations:** I Department of Life Sciences. University of Parma. Parma, Italy; II Cancer Registry and Environmental Epidemiology Unit. Fondazione IRCCS Istituto Nazionale dei Tumori. Milano, Italy; III Department of Public Health. Experimental and Forensic Medicine. Occupational Medicine Unit. University of Pavia. Pavia, Italy; IV Service for Occupational Safety and Prevention. Local Health Unit (ASL). Brescia, Italy; V Evaluative Epidemiology Unit. Fondazione IRCCS Istituto Nazionale dei Tumori. Milano, Italy

**Keywords:** Child, Carcinogens, Environmental Pollution, adverse effects, Neoplasms, prevention & control, Environmental Exposure, Environmental Monitoring

## Abstract

Improvements in the health standards of developed and developing societies depend primarily on the relationships between economy and environment. Recent long-term changes in the chemical composition of man-made environments may be linked to changes in the biology of human beings. Here we argue that children are at the greatest risk of being affected by the dangerous effects of these changes, with particular reference to cancer. The concept of cancer risk must be extended to new contexts. Considering the increasing rates of chemical pollution and its spreading in the environment, we illustrate a proposal aiming to protect the human health, in an intra- and intergenerational perspective. A surveillance system of occupational and residential exposures should be implemented to prevent cancer risk in embryos and children.

## LOOKING TO THE FUTURE

The economist Amartya Sen – Nobel Prize laureate in 1998 – has stated that health is an essential value of human life, and the opportunity to receive health care is a primary sign of our civilization^13^. According to Sen, investing in public health strategies is not just an ethical investment but rather an investment in a wholesome society. Health, with education, is a keystone of human capital, which is a prerequisite for developing a sound economy; in other words, it is an investment for young persons and new generations. The causes of death and disability on a global scale, therefore, need to be clarified, mapped, and prevented as part of a major effort to achieve a high standard of health and wellbeing everywhere. Here we argue that children are at the greatest risk of being affected by the dangerous effects of toxic chemicals, and a surveillance system of occupational and residential exposures with a transgenerational perspective should be implemented.

## LEARNING FROM THE PAST

Improvements in the health standards of our societies depend primarily on substantial changes in social attitudes (dietary, lifestyles etc.) and – of particular interest here – on the relationship between economic patterns and environment. Some proofs of the environmental effects on human health come from anthropological and historical research. Field studies conducted among indigenous groups living in different regions of Latin America have shown that, when the integrity of the environment is preserved, the local populations enjoy good health indicators^11^. Conversely, disruption and commercial exploitation of natural resources cause severe deterioration of environmental matrices and loss of biodiversity. In human populations living in these environments, overall health conditions were worse, because the loss of environmental integrity induces profound changes both in ecologic and socioeconomic systems^1^ ^,^ ^12^.

Recent long-term changes in the chemical composition of man-made environments may be linked to changes in the biology of living beings, including humans^16^. Over the last half century, ecologists and toxicologists have directed their attention to the environmental hazard resulting from the widespread release of synthetic compounds by human activities. They have focused specially on the traditional and linear dose-response effects of acute and chronic exposures, with particular reference to the work setting. The issue of low-dose exposure to synthetic chemicals in living beings was overlooked until zoologists began to study the exposure to chemicals with hormone-like effects, laying the foundation of the wildlife-human connection^7^. So far, little attention has been paid to how changes in the chemical composition of the environment affect the human health by altering physiological processes involved in sexual reproduction and biological development. However, with the emergent epigenetic approach in environmental health research, it is now possible to argue that environmental pollutants can affect the human biology and health in a variety of unconventional ways.

Considering the increasing rates of chemical pollution and its spreading in environmental matrices, here we make a proposal aiming to protect the human health, in an intra- and intergenerational perspective^14^. As the global scale of the ecological and human health impact becomes ever clearer, the current economic model needs extensive transformation and urgent political action^9^. International data on the increasing burden of cancer should be evaluated by considering our economic interaction with the environment and the limited effectiveness of the current primary prevention strategies to fight cancer and other developmental diseases on a global scale.

## THE INCREASE OF CHILDHOOD CANCER

Around the world, cancer is expected to be an increasingly important cause of morbidity and mortality in the next few decades. The challenges of reducing the burden of this disease are enormous, especially when combined with population aging^17^. However, aging alone only partially accounts for the general increase in cancer incidence and it obviously cannot be accounted for at all in children and young persons. Some studies suggest there has been an evident increase of cancer incidence in childhood and adolescence during the past decades, with a recent acceleration of this trend^15^.

To explain partially these observations, some authors have suggested that dangerous exposures may occur during early life, or even before; in other words, the development of cancer might be the effect of exposure to environmental risk factors in critical periods of development *in utero*, in childhood or in adolescence^4^. Developmental immaturity leaves embryos and children more vulnerable and susceptible than adults to environmental toxicants. Many compounds, such as pesticides, household chemicals, traffic emissions, polycyclic aromatic hydrocarbons, persistent organic pollutants, and other agents that affect environmental quality may act as harmful factors whose pathological effects might appear early or later in life^2^ ^,^ ^18^.

Advances in genome-wide technologies provide evidence that early exposure to toxic chemicals can not only damage the DNA but also affect cell-to-cell signalling. Leading to perturbed developmental processes, toxic chemicals such as endocrine disruptors can promote increased susceptibility to teratogenesis, chronic diseases, and malignancies later in life^3^ ^,^ ^6^. In addition, environmental factors can become embedded in the biology of human germ cells because of durable and heritable epigenetic alterations that change the patterns of genetic expression in individuals^6^ ^,^ ^8^ ^,^ ^10^ ^,^ ^16^.

Further risk factors, such as ionizing radiation and inherited (genetic) alterations, are thought to account for 5%-10% of childhood cancers, while other causes related to infectious agents explain a very marginal proportion of the burden of the disease worldwide^5^.

Although our knowledge of the etiological factors of childhood tumours is partial, the literature indicates that several aspects are clear:

- data on international differences in incidence and risk factors suggest that most non communicable diseases, including childhood cancers, are likely to have developmental, nongenetic causes^3^ ^,^ ^5^;
- globalization and economic dysregulation have caused rapid changes in lifestyles and in the environmental settings where human beings live^9^ ^,^ ^16^, which is becoming increasingly important to childhood health;
- as childhood cancer can depend on environmental exposures in the early periods of individual life – including preconception, gestation, and post-birth –, it becomes ever clearer that a different time horizon of studies is needed to cover both the full lifetime of individuals and the transgenerational cycle^3^ ^,^ ^4^.

## OUR PROPOSAL

Here we highlight the need to establish a monitoring network of researchers and public health practitioners to investigate the environmental and occupational determinants of childhood cancer and develop ecological and public health strategies.

Prompt action is needed. Surveillance of childhood cancer should be scheduled periodically using population-based cancer registries. Preventive and precautionary policies based on the eco-geographical distribution of childhood cancer should be a pre-requisite of the effort to protect the new generations from the environmental effects of poor social and economic dynamics. Occupational physicians, human ecologists, epidemiologists, public health practitioners, urbanists, agronomists, and other professionals must grasp this opportunity to tackle childhood cancer in an interdisciplinary way.

A monitoring network would work on a set of challenging issues, including issues related to:

1. residential risk: the influence of moderate to high levels of air, water, and soil pollution on childhood cancer risk;
2. household risk (indoor and outdoor): the influence of moderate to high levels of drinking water, food, home, and garden and backyard pollution on childhood cancer risk;
3. occupational risk (parental): the role of moderate to high chemical exposure in occupational settings in the assessment of cancer risk in workers and their offspring;
4. nonoccupational risk (parental): the role of the intensity, timing, and route of nonoccupational exposure in the assessment of cancer risk in adults and their offspring;
5. correlation of markers: the relationship between markers of ecological integrity and cancer incidence;
6. precaution and primary prevention: the use of reviews and meta-analyses of scientific data on childhood cancer in different contexts (occupational and non-occupational) to promote policies on risk reduction and/or elimination.

## CONCLUSION

We suggest here that a transnational effort to explore parental and childhood exposure to environmental contamination suspected of causing cancer could produce major benefits. This exposure should be evaluated using new biomedical and ecological information and new tools to implement public health policies and measures for primary prevention. The types of exposure briefly mentioned here are critical because of their potential to cause cancer as well as harm embryos, infants, and children in other ways. These exposures can be prevented, thus an effort is mandatory.

Strategies to monitor parental occupational cancer and reduce its burden would draw on advanced know-how from different scientific fields. The first step would be to set up a network of European and American researchers and professionals to develop skills and acquire experience on childhood cancer and environmental integrity, for detecting, mapping, and managing the risk.
